# Early Access to Testosterone Therapy in Transgender and Gender-Diverse Adults Seeking Masculinization: A Randomized Clinical Trial

**DOI:** 10.1001/jamanetworkopen.2023.31919

**Published:** 2023-09-07

**Authors:** Brendan J. Nolan, Sav Zwickl, Peter Locke, Jeffrey D. Zajac, Ada S. Cheung

**Affiliations:** 1 Department of Endocrinology, Austin Health, Heidelberg, Victoria, Australia; 2 Trans Health Research Group, Department of Medicine (Austin Health), University of Melbourne, Heidelberg, Victoria, Australia; 3 Equinox Gender Diverse Clinic, Thorne Harbour Health, Abbotsford, Victoria, Australia

## Abstract

**Importance:**

Testosterone treatment is a necessary component of care for some transgender and gender-diverse individuals. Observational studies have reported associations between commencement of gender-affirming hormone therapy and improvements in gender dysphoria and depression, but there is a lack of data from randomized clinical trials.

**Objective:**

To assess the effect of testosterone therapy compared with no treatment on gender dysphoria, depression, and suicidality in transgender and gender-diverse adults seeking masculinization.

**Design, Setting, and Participants:**

A 3-month open-label randomized clinical trial was conducted at endocrinology outpatient clinics and primary care clinics specializing in transgender and gender-diverse health in Melbourne, Australia, from November 1, 2021, to July 22, 2022. Participants included transgender and gender-diverse adults aged 18 to 70 years seeking initiation of testosterone therapy.

**Interventions:**

Immediate initiation of testosterone commencement (intervention group) or no treatment (standard care waiting list of 3 months before commencement). This design ensured no individuals would be waiting longer than the time to standard care.

**Main Outcomes and Measures:**

The primary outcome was gender dysphoria, as measured by the Gender Preoccupation and Stability Questionnaire. Secondary outcomes included the Patient Health Questionnaire-9 (PHQ-9) to assess depression and the Suicidal Ideation Attributes Scale (SIDAS) to assess suicidality. Questionnaires were undertaken at 0 and 3 months. The evaluable cohort was analyzed.

**Results:**

Sixty-four transgender and gender-diverse adults (median [IQR] age, 22.5 [20-27] years) were randomized. Compared with standard care, the intervention group had a decrease in gender dysphoria (mean difference, −7.2 points; 95% CI, −8.3 to −6.1 points; *P* < .001), a clinically significant decrease in depression (ie, change in score of 5 points on PHQ-9; mean difference, −5.6 points; 95% CI, −6.8 to −4.4 points; *P* < .001), and a significant decrease in suicidality (mean difference in SIDAS score, −6.5 points; 95% CI, −8.2 to −4.8 points; *P* < .001). Resolution of suicidality assessed by PHQ-9 item 9 occurred in 11 individuals (52%) with immediate testosterone commencement compared with 1 (5%) receiving standard care (*P* = .002). Seven individuals reported injection site pain/discomfort and 1 individual reported a transient headache 24 hours following intramuscular administration of testosterone undecanoate. No individual developed polycythemia.

**Conclusions and Relevance:**

In this open-label randomized clinical trial of testosterone therapy in transgender and gender-diverse adults, immediate testosterone compared with no treatment significantly reduced gender dysphoria, depression, and suicidality in transgender and gender-diverse individuals desiring testosterone therapy.

**Trial Registration:**

ANZCTR Identifier: ACTRN1262100016864

## Key Points

**Question:**

What is the effect of testosterone therapy compared with no treatment on gender dysphoria, depression, and suicidality in transgender and gender-diverse adults seeking masculinization?

**Findings:**

In this 3-month open-label randomized clinical trial of 64 transgender and gender-diverse adults, there was a statistically significant decrease in gender dysphoria in individuals with immediate compared with delayed initiation of testosterone therapy. A clinically significant decrease in depression and a decrease in suicidality also occurred with immediate testosterone therapy.

**Meaning:**

The findings of this trial suggest that testosterone therapy significantly decreases gender dysphoria, depression, and suicidality in transgender and gender-diverse individuals desiring testosterone therapy.

## Teaser

This randomized clinical trial examines the effects of immediate initiation of testosterone therapy on gender dysphoria among transgender or gender-diverse individuals seeking masculinization compared with those who who experience a 3-month waiting period.

## Introduction

Transgender and gender-diverse individuals (including those with a binary and/or nonbinary gender identity) are a highly marginalized group. Some transgender and gender-diverse individuals experience psychological distress due to the incongruence between their gender identity and sex presumed at birth, known as gender dysphoria. There are also disproportionate rates of mental health comorbidities among transgender and gender-diverse individuals compared with the general population.^1^ Seventy-three percent of transgender and gender-diverse individuals reported a history of depression and 67% reported a history of anxiety in a recent online survey from Australia.^2^ In addition, 43% of individuals in this analysis reported a previous suicide attempt and similar statistics are seen worldwide.^3,4,5^

Some transgender and gender-diverse individuals seek commencement of gender-affirming hormone therapy (GAHT) to permit development of physical characteristics to align with their gender identity. For individuals desiring masculinization, standard doses of parenteral or transdermal testosterone used to treat hypogonadal cisgender men are recommended in consensus guidelines.^6,7,8^

Commencement of GAHT has been associated with reduced gender dysphoria and depression in cross-sectional and uncontrolled prospective studies.^9^ However, data are currently limited, particularly examining the influence of GAHT on suicidality. Due to ethical issues associated with withholding commencement of testosterone therapy in transgender individuals, to our knowledge, no randomized clinical trial has been performed.

There has been a substantial increase in the number of transgender and gender-diverse individuals seeking GAHT^10,11^; however, many report that accessing the pathway to GAHT is too difficult.^2^ Such barriers to accessing health care have been associated with suicide attempts in the transgender and gender-diverse population.^12^ It is therefore imperative to evaluate the effect of earlier access to GAHT on gender dysphoria and mental health outcomes in this at-risk population.

We undertook a 3-month open-label randomized clinical trial to assess the effect of testosterone therapy vs no treatment on gender dysphoria, depression, and suicidality in transgender and gender-diverse individuals seeking initiation of testosterone therapy. We hypothesized that there would be a reduction in gender dysphoria, depression, and suicidality in transgender and gender-diverse people treated with testosterone therapy compared with transgender and gender-diverse people not receiving testosterone therapy.

## Methods

### Study Design

We conducted a 3-month open-label randomized clinical trial in transgender and gender-diverse individuals seeking initiation of testosterone therapy. Following clinical assessment and establishment of the person’s capacity to initiate testosterone administration using the informed consent model of care,^13^ individuals were randomized to immediate testosterone commencement (intervention group) within 1 week of the first study visit or no treatment (commencement of testosterone following a standard care waiting list of 3 months). This design ensured no individuals would be waiting longer than standard care. Participants did not have formal mental health assessment if deemed suitable to commence treatment using the informed consent model of care. Participants provided written informed consent. Participants completed questionnaires evaluating gender dysphoria, depression, and suicidality at baseline and 3 months. The trial was approved by the Human Research Ethics Committee at Austin Health and lesbian, gay, bisexual, transgender, intersex community organization Thorne Harbour Health Community Research Endorsement Panel. The trial was registered with the Australian New Zealand Clinical Trials Registry. We followed the Consolidated Standards of Reporting Trials (CONSORT) reporting guideline. The CONSORT flow diagram is shown in the Figure. The protocol can be found in Supplement 1.

**Figure.**
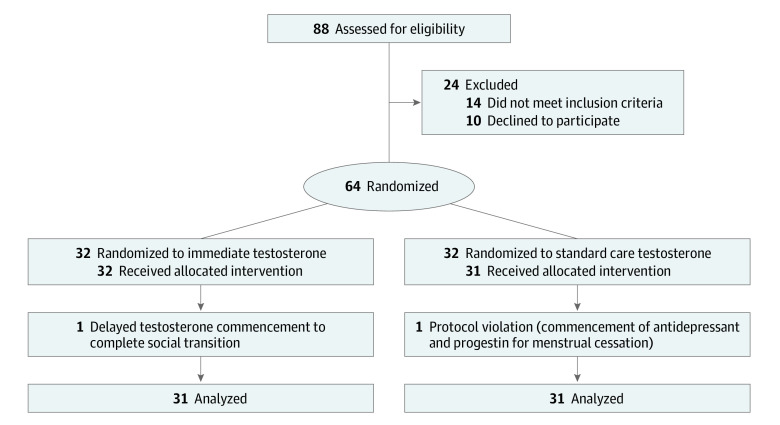
Study Participants

### Participants and Setting

Participants were transgender and gender-diverse individuals aged 18 to 70 years seeking initiation of testosterone therapy with no previous testosterone treatment. Individuals were recruited from endocrinology outpatient clinics and primary care clinics specializing in transgender and gender-diverse health in Melbourne, Australia, from November 1, 2021, to July 22, 2022. Individuals were excluded if they had a contraindication to receipt of testosterone, including androgen-dependent carcinoma, known cancer, hypersensitivity to the active substance or to any of the excipients, polycythemia (defined as hematocrit >50% [to convert to proportion of 1.0, multiply by 0.01]^6^) at baseline, known liver tumor, uncontrolled hypertension (blood pressure >160/90 mm Hg despite antihypertensive medication), uncontrolled untreated sleep apnea, severe disturbance in kidney function (estimated glomerular filtration rate <30 mL/min), recent (<6 months) cardiac event or major cardiac insufficiency (New York Heart Association performance status >2), and coagulation disorders; previous testosterone treatment; substantial mental health conditions compromising the ability to provide informed consent (individuals with a history of schizophrenia or posttraumatic stress disorder are more likely to be referred for formal mental health assessment^13^); or use of medications for antiplatelet or anticoagulant therapy. Participants were assessed before enrolling in the study based on Version 2.0 of the Protocols for the Initiation of Hormone Therapy for Trans and Gender-Diverse Patients endorsed by the Australian Professional Association for Trans Health.^14^

### Intervention

Testosterone therapy consisted of administration of intramuscular testosterone undecanoate, 1000 mg, at weeks 0 and 6, or transdermal testosterone, 1% (12.5 mg/actuation), gel 4 pump actuations daily. Participants were able to choose between these formulations as per standard care.^8^ There was no dose titration during the study.

### Outcomes

Our primary outcome was gender dysphoria as measured by the Gender Preoccupation and Stability Questionnaire (GPSQ).^15^ The GPSQ has been validated in Australia for use as a tool to measure gender dysphoria in transgender and gender-diverse individuals^15^ and is able to evaluate the effectiveness of gender-affirming interventions in binary and nonbinary transgender and gender-diverse individuals. The instrument includes 14 multiple-choice questions designed to measure the extent of an individual’s preoccupation with gender and the stability of their own gender identity over the past 2 weeks.^15^ Each question has 5 possible answers, corresponding to a score of 1 to 5, with higher scores indicating higher levels of gender dysphoria. Total scores were calculated by taking the summation of all values. Total scores greater than or equal to 28 are considered highly suggestive of clinical gender dysphoria, and a change in score of 11 points or more indicates a change in the degree of gender dysphoria.^15^

Depression was measured using Patient Health Questionnaire-9 (PHQ-9).^16^ The questionnaire includes 9 multiple-choice questions assessing the frequency of the actual 9 criteria upon which the diagnosis of *Diagnostic and Statistical Manual of Mental Disorders,4th ed,* depressive disorders was based over the previous 2 weeks. Each question has 4 possible answers, corresponding to a score of 0 to 3, with higher scores indicating higher depression severity. Total scores were calculated by taking the summation of all values. A score of 0 to 4 indicates minimal depression; 5 to 9, mild depression; 10 to 14, moderate depression; 15 to 19, moderate to severe depression; and 20 to 27, severe depression. A change in PHQ-9 score of 5 points is considered clinically significant.^17^

Suicidality was measured using the Suicidal Ideation Attributes Scale (SIDAS).^18^ This questionnaire was initially validated in a cohort of Australian adults. The SIDAS is a 5-item scale assessing the frequency, controllability, closeness to attempt, distress, and interference with daily activities on 0- to 10-point scales over the past month. Total scores were calculated by taking the summation of all values. A score of 0 indicates no suicidal ideation, scores of 1 to 20 indicate low ideation, and scores greater than or equal to 21 indicate high risk of suicide behavior.^18^

Serum total testosterone concentration was determined using an immunoassay (Beckman Coulter Unicel DXI 800 analyzer, with coefficient of variation 5.5% at total testosterone concentrations of 645.5 ng/dL and 11.4% at 89.3 ng/dL [to convert to nanomoles per liter, multiply by 0.0347]; interassay coefficient of variation 7.8%; Beckman Coulter Inc).

### Sample Size

Power calculation was based on the primary end point of gender dysphoria, using change in GPSQ score over 3 months following commencement of testosterone therapy, from preliminary data from our prospective controlled study.^19^ The sample size required with a level of significance of .05 and power of 0.9 when the mean (SD) GPSQ score decreased from 41 (7) to 35 (7) after 3 months of testosterone, and assumption that the GPSQ would not change in individuals allocated to standard care, was 29 per group. Our recruitment target was 37 participants per group to allow for 20% attrition.

### Randomization

Individuals were randomized with equal probability to the 2 arms using permuted blocks with a block size of 4 stratified by the median age for individuals newly commencing testosterone in our clinic (26; IQR, 22-31 years). Permuted block randomization was generated using Stata, version 17.0 software (StataCorp LLC).

### Statistical Analysis

Descriptive statistics at baseline are presented as mean (SD) or median (IQR) as appropriate for continuous variables and frequency (percentage) for categorical variables. Analysis of covariance was used to estimate the mean difference and corresponding 95% CI between the intervention group and the standard care group, adjusted for the corresponding measure at baseline.^20^ Data were checked to ensure there was no violation of the assumptions of linearity and homogeneity of the regression slopes. Plots of the residuals were checked for violations of normality. The proportion of participants with clinically significant changes in GPSQ and PHQ-9 scores were compared using χ^2^ tests. A 2-tailed significance level of *P* < .05 was used. Statistical analyses were performed using Stata, version 17.0 software.

## Results

Sixty-four transgender and gender-diverse individuals (median [IQR] age, 22.5 [20-27] years) were enrolled. Eighty-eight individuals were invited to participate; 12 individuals were excluded due to current or previous testosterone treatment, 2 were excluded due to age younger than 18 years, and 10 declined to participate (Figure). One individual in the intervention group was excluded after delaying testosterone commencement, and an individual in the standard care group was excluded due to commencement of an antidepressant and progestin therapy for menstrual suppression. Recruitment was stopped after the sample size calculation of 29 completers per group was reached. Baseline clinical characteristics are reported in Table 1, and baseline GPSQ, PHQ-9, and SIDAS scores are reported in Table 2.

**Table 1.** Baseline Characteristics

Characteristic	No. (%)	
	Intervention (n = 31)	Standard care (n = 31)
Age, median (IQR), y	23 (20-28)	22 (20-27)
Gender identity		
Binary	17 (55)	15 (48)
Nonbinary	14 (45)	16 (52)
Comorbidities		
Depression	23 (74)	18 (58)
Anxiety	20 (65)	20 (65)
ASD	8 (26)	4 (13)
ADHD	8 (26)	6 (19)
Residence		
Metropolitan	23 (74)	25 (81)
Rural/remote	8 (26)	6 (19)
Employment status		
Employed/student	23 (74)	26 (84)
Unemployed	8 (26)	5 (16)
Total testosterone, mean (SD), ng/dL	37.5 (20.2)	25.9 (14.4)

Abbreviations: ADHD, attention deficit/hyperactivity disorder; ASD, autism spectrum disorder.

SI conversion: To convert testosterone to nanomoles per liter, multiply by 0.0347.

**Table 2.** Change From Baseline to Month 3 in Individuals Receiving Immediate Commencement Testosterone Compared With Those Receiving Standard Care

Parameter	Mean (SD)		Mean difference (95% CI)^a^
	Intervention (n = 31)	Standard care (n = 31)	
0 mo	46.2 (6.0)	44.0 (6.8)	
3 mo	38.0 (6.2)	43.4 (7.9)	−7.2 (−8.3 to −6.1)
0 mo	15.2 (4.7)	14.1 (6.0)	
3 mo	8.6 (5.2)	13.4 (6.3)	−5.6 (−6.8 to −4.4)
0 mo	12.0 (8.5)	11.6 (10.0)	
3 mo	5.6 (7.3)	11.8 (11.9)	−6.5 (−8.2 to −4.8)

^a^ The mean difference and 95% CI refer to the between-group difference over 3 months adjusted for the corresponding measure at baseline. All differences were significant at *P* < .001.

Testosterone formulations were intramuscular testosterone undecanoate (n = 19) and transdermal testosterone, 1%, gel (n = 12) in the intervention group and intramuscular testosterone undecanoate (n = 18) and transdermal testosterone, 1%, gel (n = 13) in the standard care group. Baseline mean (SD) serum total testosterone concentration was 37.5 (20.2) ng/dL in the intervention group and 25.9 (14.4) ng/dL in the standard care group, and 391.9 (239.2) ng/dL at month 3 in the intervention group.

### Gender Dysphoria

The mean (SD) GPSQ score of the cohort at baseline was 45.1 (6.4), with all individuals reporting clinically significant levels of gender dysphoria (GPSQ score >28). Compared with controls, in individuals receiving testosterone, there was a significant reduction in GPSQ score over 3 months’ follow-up (mean difference, −7.2 points; 95% CI, −8.3 to −6.1 points; *P* < .001) (Table 2). A clinically significant improvement greater than or equal to 11 points in the GPSQ score was reported in 12 individuals (39%) treated with testosterone and in no individuals continuing standard care (*P* < .001).

### Depression

The overall baseline PHQ-9 score was 0 to 4 in 3 individuals (5%), 5 to 9 in 9 individuals (15%), 10 to 14 in 15 individuals (24%), 15 to 19 in 21 individuals (34%), and 20 to 27 in 14 individuals (23%). Compared with the control participants, in individuals receiving testosterone, there was a significant decrease in PHQ-9 scores over 3 months’ follow-up (mean difference, −5.6 points; 95% CI, −6.8 to −4.4 points; *P* < .001) (Table 2). A clinically significant improvement greater than or equal to 5 points in the PHQ-9 score was reported in 19 individuals (61%) treated with testosterone and 4 individuals (13%) continuing standard care (*P* < .001).

### Suicidality

The overall baseline SIDAS score was 0 in 12 individuals (19%), 1 to 20 in 41 individuals (66%), and 21 to 50 in 9 individuals (15%). Compared with the control participants, in individuals receiving testosterone, there was a significant reduction in the SIDAS score over 3 months’ follow-up (mean difference, −6.5 points; 95% CI, −8.2 to −4.8 points; *P* < .001) (Table 2).

In individuals with suicidality at baseline as assessed by PHQ-9 item 9 (“Over the last 2 weeks, how often have you been bothered by thoughts that you would be better off dead, or thoughts of hurting yourself in some way?”), resolution occurred in 11 individuals (52%) with immediate testosterone commencement compared with 1 individual (5%) in standard care (*P* = .002).

### Adverse Events

Seven individuals reported injection site pain/discomfort and 1 individual reported a transient headache 24 hours following intramuscular administration of testosterone undecanoate. No individual developed polycythemia.

## Discussion

In this 3-month open-label randomized clinical trial of transgender and gender-diverse individuals seeking initiation of testosterone, we found a significant reduction in gender dysphoria, depression, and suicidality following testosterone commencement compared with no treatment.

### Gender Dysphoria

Changes in gender dysphoria following commencement of GAHT have been quantified in a small number of studies.^19,21,22^ To date, 1 prospective controlled trial evaluated changes in gender dysphoria and quality of life following commencement of testosterone therapy, compared with a control group of cisgender women.^19^ Consistent with the current analysis, this study reported a decrease in gender dysphoria as measured by GPSQ score over 6 months. Similarly, 2 uncontrolled observational studies reported improvements in gender dysphoria following commencement of GAHT as assessed by the Gender Identity/Gender Dysphoria questionnaire^21^ and the Utrecht Gender Dysphoria Scale.^22^

In the current analysis, the mean GPSQ score decreased −7.2 points (95% CI, −8.3 to −6.1 points; *P* < .001). This is consistent with a previous prospective controlled trial^19^ but less than the clinically significant change of −11 points. This is likely related to the short-term follow-up, which is insufficient for the development of masculinizing physical characteristics and associated reductions in gender dysphoria. Despite this, 12 individuals (39%) in the intervention group had a clinically significant decrease greater than or equal to 11 points in the GPSQ following initiation of testosterone therapy.

### Depression

Transgender and gender-diverse individuals have disproportionately higher rates of depression compared with the general population,^10^ with the highest rates of depression reported in transgender people desiring commencement of hormone therapy but unable to use or access them.^23^ Most cross-sectional^21,24^ and longitudinal^25,26,27,28^ studies of 6 to 18 months of testosterone therapy have reported lower depression scores in transgender and gender-diverse adults after initiation of testosterone therapy. Notably, these longitudinal studies were uncontrolled^25,26,28^ or compared with a cisgender female control group.^27^ Several questionnaires have been used to screen for depression in these analyses, including the Zung Self-Rating Depression Score,^25^ Hospital Anxiety and Depression Scale,^26,28^ and Beck Depression Inventory.^27^

The PHQ-9 has been used to assess depression in several analyses. Consistent with our findings, a recent 12-month prospective observational study using the PHQ-9 in transgender and gender-diverse youth reported 60% lower odds of depression and 73% lower odds of suicidality in transgender and gender-diverse youth treated with puberty blockers or GAHT, compared with those who had not yet initiated treatment.^29^ An uncontrolled longitudinal study in transgender and gender-diverse youth also reported a decrease in the PHQ-9 score following commencement of GAHT.^30^ To our knowledge, the current analysis is the first randomized clinical trial to include a transgender and gender-diverse control group randomized to no treatment.

### Suicidality

The transgender and gender-diverse community has higher rates of suicide attempts and suicide compared with the general population.^31^ Large observational studies from the Netherlands reported suicide rates higher than the expected Dutch population average.^32,33^ In Australia, 43% of respondents reported a previous suicide attempt in an online survey enrolling transgender and gender-diverse participants.^2^ Similar findings have been shown globally.^3,4^ Note that being transgender or gender-diverse is not a mental illness and transgender and gender-diverse people are not inherently prone to suicide risk because of their gender identity but rather at higher risk because of widespread discrimination, abuse, and marginalization in society.^34,35^

Cross-sectional analyses have evaluated the association of GAHT with suicide with discrepant results. One study of 380 transgender and gender-diverse individuals from Canada reported that individuals undergoing GAHT were about half as likely to have considered suicide over the past year, compared with those who desire GAHT.^36^ However, these findings were not replicated in a recent analysis from Australia,^35^ potentially due to other confounding factors that influence mental health.

The SIDAS has previously been used to assess suicidality in one online survey enrolling 382 transgender and gender-diverse individuals from Australia and New Zealand.^37^ In this study, 40% of the participants scored greater than or equal to 21, indicating a high risk of suicidal behavior. SIDAS scores greater than or equal to 21 were associated with higher distress and lower social support, but the authors did not report access to GAHT.

We also report a significant reduction in suicidality between the intervention and standard care groups as assessed by item 9 of the PHQ-9. The response to PHQ-9 item 9 has been found to predict subsequent suicide attempt and suicide death in the general population.^38^

### Limitations

There are several limitations to this analysis. First, this study had a short follow-up period of 3 months, so we were unable to establish the long-term impact of testosterone on gender dysphoria, depression, or suicidality. This short period was designed for participant acceptability and feasibility so that transgender and gender-diverse participants would not be disadvantaged by waiting longer than standard care waiting times of 3 months for an initial consultation. Second, participants were not blinded to their intervention group. Therefore, it is possible that the effect of testosterone or patient knowledge of treatment has been evaluated. However, randomization of participants to no treatment or placebo over longer follow-up is unethical, particularly given preexisting barriers to accessing GAHT.^2^

Total testosterone levels were measured by immunoassay rather than by liquid chromatography/tandem mass spectrometry. However, testosterone concentrations were measured to confirm the increase of testosterone concentrations in the intervention group and a large magnitude of change was expected. The quality-controlled immunoassay routinely used in our hospital for clinical decision-making was thought to be appropriate for this purpose.

Although we used the locally developed GPSQ for our primary outcome of gender dysphoria, this tool has not been validated to measure longitudinal changes in gender dysphoria. Similarly, the SIDAS has not been validated in transgender and gender-diverse cohorts. We did not quantify other factors known to influence mental health, including social support or discrimination. Participants were given the option of 2 testosterone formulations as per standard care in Australia. However, these formulations may not be bioequivalent. Finally, the trial participants were predominantly of White race and based in metropolitan areas in Australia, so findings might not be generalizable to other cohorts.

Despite these limitations, to our knowledge, this is the first randomized clinical trial of transgender and gender-diverse adults desiring commencement of testosterone therapy comparing individuals initiating testosterone with a control group of transgender and gender-diverse individuals randomized to no treatment.

## Conclusions

This open-label randomized clinical trial supports the use of testosterone therapy to significantly reduce gender dysphoria, depression, and suicidality in transgender and gender-diverse adults desiring commencement of testosterone therapy. These findings have critical implications for service access and delivery to ensure timely access to gender-affirming hormone therapy.

## Notes

Supplement 1.
Trial Protocol

Supplement 2.
Data Sharing Statement

## References

[1] Reisner SL, Poteat T, Keatley J, . Global health burden and needs of transgender populations: a review. Lancet. 2016;388(10042):412-436. doi:10.1016/S0140-6736(16)00684-X 27323919 PMC7035595

[2] Bretherton I, Thrower E, Zwickl S, . The health and well-being of transgender Australians: a national community survey. LGBT Health. 2021;8(1):42-49. doi:10.1089/lgbt.2020.0178 33297824 PMC7826417

[3] Clements-Nolle K, Marx R, Katz M. Attempted suicide among transgender persons: the influence of gender-based discrimination and victimization. J Homosex. 2006;51(3):53-69. doi:10.1300/J082v51n03_04 17135115

[4] Maguen S, Shipherd JC. Suicide risk among transgender individuals. Psychol Sex. 2010;1(1):34-43.

[5] Chen R, Zhu X, Wright L, . Suicidal ideation and attempted suicide amongst Chinese transgender persons: national population study. J Affect Disord. 2019;245:1126-1134. doi:10.1016/j.jad.2018.12.011 30699856

[6] Hembree WC, Cohen-Kettenis PT, Gooren L, . Endocrine treatment of gender-dysphoric/gender-incongruent persons: an Endocrine Society clinical practice guideline. J Clin Endocrinol Metab. 2017;102(11):3869-3903. doi:10.1210/jc.2017-01658 28945902

[7] Coleman E, Radix AE, Bouman WP, . Standards of care for the health of transgender and gender diverse people, version 8. Int J Transgend Health. 2022;23(suppl 1):S1-S259. doi:10.1080/26895269.2022.2100644 36238954 PMC9553112

[8] Cheung AS, Wynne K, Erasmus J, Murray S, Zajac JD. Position statement on the hormonal management of adult transgender and gender diverse individuals. Med J Aust. 2019;211(3):127-133. doi:10.5694/mja2.50259 31271465

[9] Baker KE, Wilson LM, Sharma R, Dukhanin V, McArthur K, Robinson KA. Hormone therapy, mental health, and quality of life among transgender people: a systematic review. J Endocr Soc. 2021;5(4):bvab011. doi:10.1210/jendso/bvab011 33644622 PMC7894249

[10] Cheung AS, Ooi O, Leemaqz S, . Sociodemographic and clinical characteristics of transgender adults in Australia. Transgend Health. 2018;3(1):229-238. doi:10.1089/trgh.2018.0019 30596151 PMC6308273

[11] Wiepjes CM, Nota NM, de Blok CJM, . The Amsterdam Cohort of Gender Dysphoria Study (1972-2015): trends in prevalence, treatment, and regrets. J Sex Med. 2018;15(4):582-590. doi:10.1016/j.jsxm.2018.01.016 29463477

[12] Romanelli M, Lu W, Lindsey MA. Examining mechanisms and moderators of the relationship between discriminatory health care encounters and attempted suicide among US transgender help-seekers. Adm Policy Ment Health. 2018;45(6):831-849. doi:10.1007/s10488-018-0868-8 29574543

[13] Spanos C, Grace JA, Leemaqz SY, . The informed consent model of care for accessing gender-affirming hormone therapy is associated with high patient satisfaction. J Sex Med. 2021;18(1):201-208. doi:10.1016/j.jsxm.2020.10.020 33249011

[14] Protocols for the initiation of hormone therapy for trans and gender diverse patients, v2.0. June 2020. Accessed February 10, 2023. https://equinoxdotorgdotau.files.wordpress.com/2021/07/protocol-for-the-initiation-of-hormone-therapy-v2-aug-2020.pdf

[15] Hakeem A, Črnčec R, Asghari-Fard M, Harte F, Eapen V. Development and validation of a measure for assessing gender dysphoria in adults: the Gender Preoccupation and Stability Questionnaire. Int J Transgenderism. 2016;17(3-4):131-140. doi:10.1080/15532739.2016.1217812

[16] Kroenke K, Spitzer RL, Williams JB. The PHQ-9: validity of a brief depression severity measure. J Gen Intern Med. 2001;16(9):606-613. doi:10.1046/j.1525-1497.2001.016009606.x 11556941 PMC1495268

[17] Löwe B, Unützer J, Callahan CM, Perkins AJ, Kroenke K. Monitoring depression treatment outcomes with the Patient Health Questionnaire-9. Med Care. 2004;42(12):1194-1201. doi:10.1097/00005650-200412000-00006 15550799

[18] van Spijker BA, Batterham PJ, Calear AL, . The suicidal ideation attributes scale (SIDAS): community-based validation study of a new scale for the measurement of suicidal ideation. Suicide Life Threat Behav. 2014;44(4):408-419. doi:10.1111/sltb.12084 24612048

[19] Foster Skewis L, Bretherton I, Leemaqz SY, Zajac JD, Cheung AS. Short-term effects of gender-affirming hormone therapy on dysphoria and quality of life in transgender individuals: a prospective controlled study. Front Endocrinol (Lausanne). 2021;12:717766. doi:10.3389/fendo.2021.717766 34394009 PMC8358932

[20] Vickers AJ, Altman DG. Statistics notes: analysing controlled trials with baseline and follow up measurements. BMJ. 2001;323(7321):1123-1124. doi:10.1136/bmj.323.7321.1123 11701584 PMC1121605

[21] Fisher AD, Castellini G, Ristori J, . Cross-sex hormone treatment and psychobiological changes in transsexual persons: two-year follow-up data. J Clin Endocrinol Metab. 2016;101(11):4260-4269. doi:10.1210/jc.2016-1276 27700538

[22] van de Grift TC, Elaut E, Cerwenka SC, . Effects of medical interventions on gender dysphoria and body image: a follow-up study. Psychosom Med. 2017;79(7):815-823. doi:10.1097/PSY.0000000000000465 28319558 PMC5580378

[23] Hyde ZDM, Tilley M, McCaul K, Rooney R, Jancey J. The first Australian national trans mental health study: summary of results. Curtin University of Technology, School of Public Health; 2013.

[24] Gómez-Gil E, Zubiaurre-Elorza L, Esteva I, . Hormone-treated transsexuals report less social distress, anxiety and depression. Psychoneuroendocrinology. 2012;37(5):662-670. doi:10.1016/j.psyneuen.2011.08.010 21937168

[25] Colizzi M, Costa R, Todarello O. Transsexual patients’ psychiatric comorbidity and positive effect of cross-sex hormonal treatment on mental health: results from a longitudinal study. Psychoneuroendocrinology. 2014;39:65-73. doi:10.1016/j.psyneuen.2013.09.029 24275005

[26] Defreyne J, T’Sjoen G, Bouman WP, Brewin N, Arcelus J. Prospective evaluation of self-reported aggression in transgender persons. J Sex Med. 2018;15(5):768-776. doi:10.1016/j.jsxm.2018.03.079 29699761

[27] Metzger NY, Boettger S. The effect of testosterone therapy on personality traits of trans men: a controlled prospective study in Germany and Switzerland. Psychiatry Res. 2019;276:31-38. doi:10.1016/j.psychres.2019.03.053 30999214

[28] Aldridge Z, Patel S, Guo B, . Long-term effect of gender-affirming hormone treatment on depression and anxiety symptoms in transgender people: a prospective cohort study. Andrology. 2021;9(6):1808-1816. doi:10.1111/andr.12884 32777129

[29] Tordoff DM, Wanta JW, Collin A, Stepney C, Inwards-Breland DJ, Ahrens K. Mental health outcomes in transgender and nonbinary youths receiving gender-affirming care. JAMA Netw Open. 2022;5(2):e220978. doi:10.1001/jamanetworkopen.2022.0978 35212746 PMC8881768

[30] Achille C, Taggart T, Eaton NR, . Longitudinal impact of gender-affirming endocrine intervention on the mental health and well-being of transgender youths: preliminary results. Int J Pediatr Endocrinol. 2020;2020:8. doi:10.1186/s13633-020-00078-2 32368216 PMC7191719

[31] Marshall E, Claes L, Bouman WP, Witcomb GL, Arcelus J. Non-suicidal self-injury and suicidality in trans people: a systematic review of the literature. Int Rev Psychiatry. 2016;28(1):58-69. doi:10.3109/09540261.2015.1073143 26329283

[32] Asscheman H, Giltay EJ, Megens JA, de Ronde WP, van Trotsenburg MA, Gooren LJ. A long-term follow-up study of mortality in transsexuals receiving treatment with cross-sex hormones. Eur J Endocrinol. 2011;164(4):635-642. doi:10.1530/EJE-10-1038 21266549

[33] Wiepjes CM, den Heijer M, Bremmer MA, . Trends in suicide death risk in transgender people: results from the Amsterdam Cohort of Gender Dysphoria study (1972-2017). Acta Psychiatr Scand. 2020;141(6):486-491. doi:10.1111/acps.13164 32072611 PMC7317390

[34] Austin A, Craig SL, D’Souza S, McInroy LB. Suicidality among transgender youth: elucidating the role of interpersonal risk factors. J Interpers Violence. 2022;37(5-6):NP2696-NP2718. doi:10.1177/0886260520915554 32345113

[35] Zwickl S, Wong AFQ, Dowers E, . Factors associated with suicide attempts among Australian transgender adults. BMC Psychiatry. 2021;21(1):81. doi:10.1186/s12888-021-03084-7 33557793 PMC7869522

[36] Bauer GR, Scheim AI, Pyne J, Travers R, Hammond R. Intervenable factors associated with suicide risk in transgender persons: a respondent driven sampling study in Ontario, Canada. BMC Public Health. 2015;15:525. doi:10.1186/s12889-015-1867-2 26032733 PMC4450977

[37] Treharne GJ, Riggs DW, Ellis SJ, Flett JAM, Bartholomaeus C. Suicidality, self-harm, and their correlates among transgender and cisgender people living in Aotearoa/New Zealand or Australia. Int J Transgend Health. 2020;21(4):440-454. doi:10.1080/26895269.2020.1795959 34993522 PMC8726598

[38] Simon GE, Rutter CM, Peterson D, . Does response on the PHQ-9 Depression Questionnaire predict subsequent suicide attempt or suicide death? Psychiatr Serv. 2013;64(12):1195-1202. doi:10.1176/appi.ps.201200587 24036589 PMC4086215

