# Supplementary material for: Early Access to Testosterone Therapy in Transgender and Gender-Diverse Adults Seeking Masculinization: A Randomized Clinical Trial
Source: JAMA Netw Open. 2023 Sep 7;6(9):e2331919. doi: 10.1001/jamanetworkopen.2023.31919 (PMC10485726; doi:10.1001/jamanetworkopen.2023.31919)
Supplement: Supplement 1. — Trial Protocol [file jamanetwopen-e2331919-s001.pdf]

PROTOCOL

A randomised controlled trial of testosterone treatment on gender dysphoria, depression, suicidality and quality of life in transgender men

Protocol Number (Mandatory field): 1
Version: 3
Date: 20/09/2021

Author/s:
Brendan J Nolan
Ada S Cheung

CONFIDENTIAL

This document is confidential and the property of Austin Health. No part of it may be transmitted, reproduced, published, or used without prior written authorization from the institution.

Statement of Compliance

This document is a protocol for a research project. This study will be conducted in compliance with all stipulation of this protocol, the conditions of the ethics committee approval, the NHMRC National Statement on ethical Conduct in Human Research (2007) and the Note for Guidance on Good Clinical Practice (CPMP/ICH-135/95).

Table of Contents

Contents

Table of Contents 2

Study Synopsis …………………………………………………………………………………….. 5

1. Glossary of Abbreviations & Terms 4

2. Study Sites 5

2.1 Study Location/s 5

3. Introduction/Background Information 6

3.1 Lay Summary..6

3.2 Introduction 6

3.3 Background information 7

4. Study Objectives 7

4.1 Hypothesis 7

4.2 Study Aims..8

4.3 Outcome Measures 8

5. Study Design..8

5.1 Study Type & Design & Schedule..8

5.2 Standard Care and Additional to Standard Care Procedures**Error! Bookmark not defined.**

5.3 Randomisation..10

5.4 Study methodology..**Error! Bookmark not defined.**

6. Study Population 10

6.1 Recruitment Procedure..10

6.2 Inclusion Criteria 11

6.3 Exclusion Criteria...11

6.4 Consent 11

7. Participant Safety and Withdrawal...11

7.1 Risk Management and Safety..11

7.2 Handling of Withdrawals .... **Error! Bookmark not defined.**

7.3 Replacements .... **Error! Bookmark not defined.**

8. Statistical Methods 12

8.1 Sample Size Estimation & Justification .... **Error! Bookmark not defined.**

8.2 Power Calculations .... **Error! Bookmark not defined.**

8.3 Statistical Methods To Be Undertaken .... **Error! Bookmark not defined.**

9. Storage of Blood and Tissue Samples 12

9.1 Details of where samples will be stored, and the type of consent for future use of samples 13

10. Data Security & Handling 13

10.1 Details of where records will be kept & How long will they be stored 13

10.2 Confidentiality and Security 13

10.3 Ancillary data .... **Error! Bookmark not defined.**

11. References 13

STUDY SYNOPSIS

(please provide a brief information)

Title:	A randomised controlled trial of testosterone treatment on gender dysphoria, depression, suicidality and quality of life in transgender men.
Short Title:	Testosterone treatment in transgender men
Design:	Randomised double-blind controlled trial
Study Centres:	Austin Health, Your Community Health, Equinox Gender Diverse Health Centre, Endocrinology Melbourne, North Eastern Urology
Hospital:	Austin Hospital and Heidelberg Repatriation Hospital
Study Question:	Does testosterone treatment reduce gender dysphoria, depression and suicidal ideation, and improve quality of life in transgender men?
Study Objectives:	To assess the influence of testosterone treatment on gender dysphoria, depression, suicidal ideation, and quality of life in transgender men newly commencing testosterone
Primary Objectives:	Gender Preoccupation and Stability (GPSQ) questionnaire
Secondary Objectives	1. Patient-Health Questionnaire-9 (PHQ-9)<br>2. Suicidal Ideation Attributes Scale (SI-DAS)<br>3. EQ-5D-5L to assess quality of life and enable health economics analyses
Inclusion Criteria:	Transgender men aged 18–70 years, newly commencing testosterone therapy.
Exclusion Criteria:	1. Contraindication to testosterone<br>2. Elevated haematocrit at baseline<br>3. History of major psychiatric disease or psychological condition that may limit understanding and compliance with study requirements<br>4. Medications for antiplatelet or anticoagulant therapy
Number of Planned Subjects:	74
Investigational product:	Testosterone undecanoate 1000mg (Reandron)<br><br>Transdermal testosterone 1% gel (Testogel)<br><br>Transdermal testosterone 5% cream (AndroForte)
Safety considerations:	Polycythaemia
Statistical Methods:	Analysis of covariance (ANCOVA) will be used to determine between group differences in the main outcome measures over time.
Subgroups:	N/A

1. GLOSSARY OF ABBREVIATIONS & TERMS

Abbreviation	Description (using lay language)
E2	Estradiol
GAHT	Gender-affirming hormone therapy
GPSQ	Gender Preoccupation and Stability Questionnaire
PHQ-9	Patient Health Questionnaire-9
SI-DAS	Suicidal Ideation Attributes Scale
T	Testosterone

2. STUDY SITES

a. STUDY LOCATION/S

Site	Address	Contact Person	Phone	Email
Austin Health	145 Studley Rd, Heidelberg 3084	Brendan Nolan	0421 479 114	bjnolan@student.unimelb.edu.au
Endocrinology Melbourne	76 Edwin Street, Heidelberg Heights	Brendan Nolan	0421 479 114	bjnolan@student.unimelb.edu.au
Endocrinology Melbourne	76 Edwin Street, Heidelberg Heights	Brendan Nolan	0421 479 114	bjnolan@student.unimelb.edu.au
North Eastern Urology	11 Martin Street, Heidelberg 3084	Ada Cheung	0403 311 850	adac@unimelb.edu.au
Equinox Gender	200 Hoodle Street,	Brendan Nolan	0421 479 114	bjnolan@student.unimelb.edu.au

Diverse Health Centre	Abbotsford 3067			
Your Community Health	300 Bell Street, Preston 3072	Brendan Nolan	0421 479 114	bjnolan@student.unimelb.edu.au

3. Introduction/Background Information

a. Lay Summary

Transgender men are treated with testosterone to align their physical appearance with their gender identity and improve mental health. Although the current evidence does show improvements in mental health, there is a lack of high-quality data in this field. High-quality data is required to potentially list "gender affirmation" as an indication for commencement of testosterone treatment with the Therapeutic Goods Administration and Pharmaceutical Benefits Scheme.

Currently, in our Austin Health gender clinic, following initial assessment for suitability of gender transition and informed consent, due to large demand for appointments, patients typically wait a 3-month period to commence testosterone therapy. This is standard care.

We will undertake a pragmatic intervention whereby after initial assessment and informed consent, we will randomise individuals to an immediate testosterone therapy compared to a delayed testosterone therapy group (which due to standard care clinic waiting times for a review appointment, is an approximately 3-month wait to commence testosterone therapy).

This project is a trial of transgender men newly commencing testosterone therapy. We aim to establish the influence of testosterone on gender dysphoria, depression, suicidality and quality of life. Men will be in our trial for 3 months and attend on 2 occasions for study assessments. Men in the trial will be closely monitored by the study doctors, and their usual Endocrinologist.

b. Introduction

Despite testosterone therapy being standard care for transgender people seeking masculinisation, there is much criticism that insufficient quality research exists to support its use (1-3). No randomised controlled trials exist and gender affirmation in transgender people is not a specific indication listed by the Therapeutic Goods Administration (TGA) nor is it a clear indication in the Pharmaceutical Benefits Scheme (PBS). Many argue that it is not ethical to do a randomised controlled trial given the "known benefits of testosterone therapy". In order to achieve gender affirmation being listed as an indication by the TGA and subsequently PBS, we will collaborate with community groups to conduct a rigorous randomised trial to demonstrate effectiveness of testosterone therapy to relieve gender dysphoria and improve quality of life. Currently, in our Austin Health gender clinic, following initial assessment for suitability of gender transition and informed consent, due to large demand for appointments, patients typically wait a 3-month period to commence testosterone therapy. This is standard care.

We therefore undertake a pragmatic intervention whereby after initial assessment and informed consent, we will randomise individuals to an immediate testosterone therapy compared to a delayed testosterone therapy group (which due to standard care clinic waiting times for a review appointment, is an approximately 3-month wait to commence testosterone therapy). Ordinarily, transgender participants would not accept being randomised to delayed

treatment, but, as this delay is currently standard care, being allocated to the immediate treatment group will be perceived as a desired benefit. This pragmatic design enables a 3-month period where we can compare transgender men with gender dysphoria who receive testosterone therapy compared to no treatment.

c. Background information

Transgender individuals (0.6-3% of the population, approximately 150,000 – 720,000 Australians), whose gender identity does not match their sex designated at birth, often experience significant distress and unease termed gender dysphoria. Gender dysphoria is often accompanied by a strong desire to align their physical characteristics with their gender identity through use of gender-affirming hormone therapy (GAHT). Transgender people in Australia are marginalized, face widespread discrimination and resultant alarming health disparity. Rates of diagnosed depression in trans Australians are over four times that of the general population and concerningly, 43% have attempted suicide (4,5). This unmet need; suicidality and depression stemming from gender dysphoria is a consequence of not being effectively treated. Despite the conservative estimate of 150,000 trans Australians (6,7), there is an almost complete absence of high quality, properly controlled research in trans health.

Testosterone therapy in transgender men

Transgender men are treated with testosterone to increase total testosterone concentration to the male (10-30 nmol/L) reference range to induce masculinisation which is associated with improved psychological functioning quality of life, depression and suicidal ideation in cohort and cross-sectional studies (8,9).

Standard doses of testosterone used to treat hypogonadal men are recommended for transgender men (3). To date, only one small (n=45) randomised trial comparing different testosterone formulations (but not to placebo) has been published in transgender men and did not find any significant difference between formulations with respect to body composition, bone or metabolic parameters (10).

Gender dysphoria

Our preliminary data from a prospective case-control study has demonstrated **decreased gender dysphoria** in transgender men following initiation of testosterone therapy but this was compared to cisgender controls and not transgender men (Figure 1). Compared to age-matched cisgender female controls (n=47), transgender men (n=27) had a significant reduction in gender dysphoria after 3 months of GAHT (mean difference -6.20 [-8.04, -4.37], p<0.001) (Figure 1). We note limitations that the controls were not people with gender dysphoria.

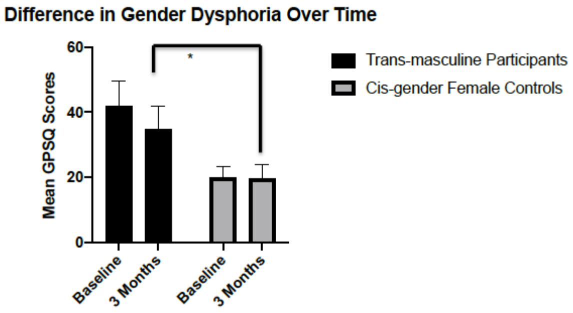

Figure 1: Difference in gender dysphoria over time. Mean (SD) GPSQ scores presented for baseline and 3 months visits. *p<0.001

4. Study Objectives

a. Hypothesis

In transgender men newly commencing testosterone therapy, testosterone treatment will: **(a)** reduce gender dysphoria; **(b)** reduce depression; **(c)** reduce suicidal ideation; and, **(d)** improve quality of life over 3 months compared to no treatment.

b. Study Aims

In a randomised controlled trial of immediate vs delayed treatment in transgender men newly commencing testosterone therapy, we aim to assess the effect of testosterone therapy compared to no treatment over 3 months on gender dysphoria, depression, suicidal ideation, and quality of life.

c. Outcome Measures

Primary endpoint:

- Gender Preoccupation and Stability Questionnaire (GPSQ) (11)

Secondary endpoints:

- Patient-Health Questionnaire-9 (PHQ-9) (12)
- Suicidal Ideation Attributes Scale (SI-DAS) (13)
- EQ-5D-5L to enable health economics analyses (14)
- Total testosterone concentration measured via immunoassay

Safety endpoint:

- Haematocrit

Measures:

Questionnaires:

Gender dysphoria will be assessed using the Gender Preoccupation and Stability Questionnaire (GPSQ), depression with the Patient-Health Questionnaire-9 (PHQ-9), suicidal ideation with the Suicidal Ideation Attributes Scale (SI-DAS), and quality of life with EQ-5D-5L. Copies of these questionnaires are attached in the appendix to this protocol.

Laboratory studies (blood tests):

Serum sex steroids [total testosterone, estradiol] will be measured by immunoassay.

5. Study Design

a. Study Type & Design & Schedule

1. Design

   Multi-centre randomised controlled trial to assess changes in gender dysphoria, depression, suicidal ideation and quality of life in transgender men newly commencing testosterone therapy (immediate) with transgender men commencing testosterone therapy in 12 weeks' time (standard care).

   As per standard care in conjunction with their treating clinicians, participants will be able to choose testosterone undecanoate 1000mg intramuscular injections, or standard dose transdermal testosterone gel or cream.

   Testosterone undecanoate will be administered at week 0, 6 and 18 weeks (Group 1) or Week 12 and 18 (Group 2). Transdermal testosterone gel or cream will be applied daily from week 0 (Group 1) or from Week 12 (Group 2).

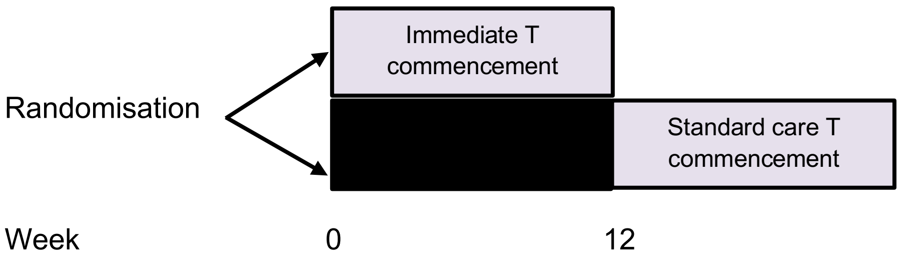

2. Data and sample handling
All collected data will be in an individually re-identifiable form. Paper information will be stored in an individual study file which will be identified only by a study number. This will be kept in a locked filing cabinet in the Department of Medicine (Austin Health). The code to match study number with identifying patient demographic details will be stored in an electronic file in a password protected area of a Department of Medicine (Austin Health) server. Other electronic data will be stored similarly, kept in a separate password protected file.

Laboratory studies will be performed at Austin Pathology or Melbourne Pathology. The results of these tests will go into the participant's Austin Health medical record and be collected separately in their study file. Blood will be discarded within 14 days.

3. Study visits and data collected
<u>Screening of potential participants that consent:</u>
   - Medical interview and review of medical file: demographic data (name, age, date of birth), and medical history and medication history sufficient to determine trial eligibility.
   - Blood tests. Collected by standard venepuncture.

<u>Visit 1 (Baseline)</u>
   - Questionnaires.

<u>Visit 2 (12 weeks after baseline visit)</u>
   - Questionnaires.
   - Blood tests.

4. Schedule, standard care and additional to standard care procedures

**Assessment/Procedure**	**Screening**	**Visit 1 (Baseline)**	**Visit 1 (12 weeks)**	**Notes**
Time for study visit	60 minutes	15 minutes	30 minutes	
Informed consent	x			
Medical interview	x			Standard care

Questionnaires		x	x	In addition to standard care
Blood Collection	x		x	9mL collected at each time point (standard care)

5. Timeframe
   We expect recruitment to be completed within 24 months (recruitment of approximately three participants per month). Final study visits will have been completed by 27 months, followed by data analysis and submission of the primary manuscript within 3 months of study closure.

6. Publication
   We anticipate the results of this trial will be published in the peer-reviewed medical literature.

b. RANDOMISATION

All participants will be randomised with aid of statistician with an equal probability to immediate commencement or standard care testosterone. Randomisation will be according to a computer-generated randomisation procedure, stratified in blocks for baseline age (<26 and ≥26-year-old), with cut-offs corresponding to medians found in our previous studies of transgender individuals. A block size of 4 is chosen, with intervention group allocation randomly permuted and balanced within blocks.

c. TESTOSTERONE

Testosterone undecanoate (Reandron™) 1000mg, transdermal testosterone 1% gel (Testogel™) or transdermal testosterone 5% cream (Androforte™) will be purchased by the participant as standard care.

Testosterone will be prescribed by study investigators and contain standard instructions regarding storage and application. Testosterone undecanoate will be administered at week 0, 6 and 18 weeks (Group 1) or Week 12 and 18 (Group 2). Transdermal testosterone gel will be applied daily from week 0 (Group 1) or from Week 12 (Group 2).

6. STUDY POPULATION

a. RECRUITMENT PROCEDURE

This trial will recruit from a population of transgender men referred to Austin Health Gender Clinic, Your Community Health, Equinox Gender Diverse Clinic and private endocrinology clinics (Endocrinology Melbourne and North Eastern Urology). Specifically, we will be recruiting transgender men who have been referred for baseline and ongoing management of hormonal therapy for gender dysphoria.

We will also take referrals of eligible transgender men from Austin Health Endocrinologists in private practice who are performing an analogous role to that of the public Gender Clinic. If not already patients of Austin Health, these participants would receive an Austin Health unit record number.

b. Inclusion Criteria

Transgender men aged 18–70 years, newly commencing testosterone therapy.

c. Exclusion Criteria

1. Contraindication to testosterone, including androgen-dependent carcinoma, known malignancy, hypersensitivity to the active substance or to any of the excipients, polycythaemia at baseline, known liver tumour, uncontrolled hypertension (>160/90mmHg despite anti-hypertensive medication), uncontrolled untreated sleep apnoea, severe disturbance in renal function (estimated glomerular filtration rate <30 ml/min), recent (<6 months) cardiac event or significant cardiac insufficiency (New York Heart Association performance status >2), coagulation disorders
2. Previous testosterone treatment
3. History of major psychiatric disease or psychological condition that may limit understanding and compliance with study requirements
4. Medications for antiplatelet or anticoagulant therapy

d. Consent

Individual informed consent will be obtained from each participant.

The invitation to participate will come after an entire medical consultation. This consultation will include an entire medical history, physical examination, and discussion regarding current treatment guidelines. Only at that point, should eligibility criteria be met, would a potential participant be asked if they would be interested in hearing about the study. Consent forms will be supplied by study investigators. Dr Cheung or Dr Nolan will obtain consent.

Based on the clinical assessments made during the initial routine consultation, patients who are revealed not to have capacity to consent would not be invited to participate. Additionally, the consent process itself will help to ensure that only patients with capacity to decide, and who voluntarily agree, will participate.

7. Participant Safety and Withdrawal

A. Risk Management and Safety

1. Possible risks associated with testosterone treatment

Testosterone is an experimental product in transgender men. It is approved by the Australian Therapeutics Goods Administration (TGA) for use in cisgender men to treat primary and secondary male hypogonadism. Possible side effects from testosterone are:

Side effect	Incidence	Severity	Duration
Polycythaemia	1 in 6	Often asymptomatic	May require dose adjustment to improve
Injection site reaction	Common	Often mild	1-3 days

Pulmonary oil microembolism	Rare (<1/1,000)	Mild to severe	Days

Any side effects would be managed at Austin Health. This would be free of charge as a public patient.

2. **Risks associated with blood tests**

Venepuncture can cause some discomfort and bruising. Infection is extremely rare following venepuncture.

3. **Psychological distress**

We do not envisage that this study will cause any participant psychological distress. If participants do experience distress, they will be encouraged to speak to the study doctors.

An appropriate level of care will be triaged by the study doctors. This might include withdrawal from the study, referral to GP or Austin Health psychologist for psychological support.

B. Handling of Withdrawals

Participants who wish to withdraw from the study or who need to be withdrawn for another reason will be reviewed by a study doctor to discuss this. They will sign the Withdrawal of Participation form. It will be clear to them throughout the trial and at that time, that withdrawal will not negatively impact their relationship with their treating doctors, or Austin Health. No additional personal or health or samples information will be collected from participants who withdraw.

C. Replacements

With the sample size of 74, the study is adequately powered to determine the primary endpoint even accounting for 20% attrition. Study recruitment is progressive so that at any time point, only a proportion of total participants will be in the intervention phase. This allows progressive recruitment to ensure the study is adequately powered, accounting for withdrawals.

8. Statistical Methods

Based on our preliminary data from a case-control study of transgender men newly commencing testosterone treatment, mean GPSQ decreased from 41±7 to 35±7 after 3 months of testosterone treatment. Therefore, a sample size of 29 per group is required to achieve power 0.9 and level of significance 0.05. With a conservative drop-out rate of 20%, a total of 74 patients will be enrolled in the study.

We plan to use analysis of covariance (ANCOVA) to determine between group differences in the main outcome measures over time, adjusted for the value at baseline. The mean difference plus 95% CI between the groups from baseline to study end will be determined, and p values <0.05 are considered statistically significant.

9. Storage of Blood and Tissue Samples

a. Details of where samples will be stored, and the type of consent for future use of samples

All laboratory studies will be performed at Austin Pathology. The results of these tests will go into the participant's Austin Health medical record and be collected separately in their study file. Blood will be discarded within 14 days. We will not store blood for future use.

10. Data Security & Handling

A. Details of where records will be kept & How long will they be stored

Paper copy information will be stored in a locked filing cabinet within a locked office located in the University of Melbourne Department of Medicine, Austin Health. Each patient will have a file that will be identified by study number only. An electronic database file will also be kept for each patient as back up and as a way to analyse this information. This will also be identified by study number only and stored on a password-protected file on a Department of Medicine Server. Paper and electronic information study files will be destroyed after 15 years.

B. Confidentiality and Security

Paper and electronic files will be only identified by study number. They will be devoid of patient identifiers including name, address, date of birth, contact details and hospital unit record number.

Paper information will be stored in a locked filing cabinet within a locked office in the Department of Medicine. Electronic information will be on a Department of Medicine server. Access to this server is tightly regulated by University of Melbourne IT services. In addition, the file will be password protected.

Data security will be the responsibility of the PI.

11. References

1. **Hembree WC, Cohen-Kettenis PT, Gooren L, Hannema SE, Meyer WJ, Murad MH, Rosenthal SM, Safer JD, Tangpricha V, T'Sjoen GG**. Endocrine Treatment of Gender-Dysphoric/Gender-Incongruent Persons: An Endocrine Society Clinical Practice Guideline. J Clin Endocrinol Metab 2017; 102:3869-3903
2. **Coleman E, Bockting, W., Botzer, M., Cohen-Kettenis, P., DeCuypere, G., Feldman, J., et al.** Standards of Care for the Health of Transsexual, Transgender, and Gender-Nonconforming People, Version 7. International Journal of Transgenderism 2011; 13:165–232
3. **Cheung AS, Wynne K, Erasmus J, Murray S, Zajac JD**. Position statement on the hormonal management of adult transgender and gender diverse individuals. Med J Aust 2019; 211:127-133
4. **Bretherton I, Thrower E, Zwickl S, Wong A, Chetcuti D, Grossmann M, Zajac JD, Cheung AS**. The health and well-being of transgender Australians: a national community survey. LGBT Health 2020;
5. **Strauss P, Cook A, Winter S, Watson V, Wright Toussaint D, Lin A**. Associations between negative life experiences and the mental health of trans and gender diverse young people in Australia: findings from Trans Pathways. Psychol Med 2020; 50:808-817

6. **Flores AR, Herman JL, Gates GJ, Brown TNT**. How many adults identify as transgender in the United States? Los Angeles, CA: The Williams Institute 2016;
7. **Zhang Q, Goodman M, Adams N, Corneil T, Hashemi L, Kreukels B, Motmans J, Snyder R, Coleman E**. Epidemiological considerations in transgender health: A systematic review with focus on higher quality data. Int J Transgender Health 2020; 21:125-137
8. **White Hughto JM, Reisner SL**. A Systematic Review of the Effects of Hormone Therapy on Psychological Functioning and Quality of Life in Transgender Individuals. Transgend Health 2016; 1:21-31
9. **Tucker RP, Testa RJ, Simpson TL, Shipherd JC, Blosnich JR, Lehavot K**. Hormone therapy, gender affirmation surgery, and their association with recent suicidal ideation and depression symptoms in transgender veterans. Psychol Med 2018; 48:2329-2336
10. **Pelusi C, Costantino A, Martelli V, Lambertini M, Bazzocchi A, Ponti F, Battista G, Venturoli S, Meriggiola MC**. Effects of three different testosterone formulations in female-to-male transsexual persons. J Sex Med 2014; 11:3002-3011
11. **Hakeem A, Črnčec, R., Asghari-Fard, M., Harte, F., & Eapen, V.** Development and validation of a measure for assessing gender dysphoria in adults: The Gender Preoccupation and Stability Questionnaire. Int J Transgenderism 2016; 17:131-140
12. **Kroenke K, Spitzer RL, Williams JB**. The PHQ-9: validity of a brief depression severity measure. J Gen Intern Med 2001; 16:606-613
13. **van Spijker BA, Batterham PJ, Calear AL, Farrer L, Christensen H, Reynolds J, Kerkhof AJ**. The suicidal ideation attributes scale (SIDAS): Community-based validation study of a new scale for the measurement of suicidal ideation. Suicide Life Threat Behav 2014; 44:408-419
14. **Herdman M, Gudex C, Lloyd A, Janssen M, Kind P, Parkin D, Bonsel G, Badia X**. Development and preliminary testing of the new five-level version of EQ-5D (EQ-5D-5L). Qual Life Res 2011; 20:1727-1736
