# Supplementary material for: Early Access to Testosterone Therapy in Transgender and Gender-Diverse Adults Seeking Masculinization: A Randomized Clinical Trial
Source: JAMA Netw Open. 2023 Sep 7;6(9):e2331919. doi: 10.1001/jamanetworkopen.2023.31919 (PMC10485726; doi:10.1001/jamanetworkopen.2023.31919)
Supplement: Supplement 2. — Data Sharing Statement [file jamanetwopen-e2331919-s002.pdf]

Data Sharing Statement

Nolan. Early Access to Testosterone Therapy in Transgender and Gender-Diverse Adults Seeking Masculinization. *JAMA Netw Open*. Published September 07, 2023. doi:10.1001/jamanetworkopen.2023.31919

Data
Data available: Yes
Data types: Deidentified participant data
How to access data: nolan.b@unimelb.edu.au
When available: With publication

Supporting Documents
Document types: None

Additional Information
Who can access the data: Researchers whose proposed use of the data has been approved
Types of analyses: For a specified purpose
Mechanisms of data availability: With a signed data access agreement
